# Effects of Ashwagandha (*Withania somnifera*) on VO_2max_: A Systematic Review and Meta-Analysis

**DOI:** 10.3390/nu12041119

**Published:** 2020-04-17

**Authors:** Jorge Pérez-Gómez, Santos Villafaina, José Carmelo Adsuar, Eugenio Merellano-Navarro, Daniel Collado-Mateo

**Affiliations:** 1 HEME Research Group, Faculty of Sport Sciences, University of Extremadura, 10003 Caceres, Spain; jorgepg100@gmail.com (J.P.-G.);; 2 Physical Activity and Quality of Life Research Group (AFYCAV), Faculty of Sport Science, University of Extremadura, 10003 Cáceres, Spain; 3 Facultad de Educación, Universidad Autónoma de Chile, Talca 3460000, Chile; emerellano@gmail.com; 4 Centre for Sport Studies, Rey Juan Carlos University, Fuenlabrada, 28943 Madrid, Spain; danicolladom@gmail.com

**Keywords:** ergogenic aids, maximum oxygen consumption, performance sports, physical fitness

## Abstract

The purpose of this study was to systematically review the scientific literature about the effects of supplementation with Ashwagandha (*Withania somnifera*) on maximum oxygen consumption (VO_2max_), as well as to provide directions for clinical practice. A systematic search was conducted in three electronic databases following the Preferred Reporting Items for Systematic Reviews and Meta-Analyses Guidelines (PRISMA). The inclusion criteria were: (a) VO_2max_ data, with means ± standard deviation before and after the supplement intervention, (b) the study was randomized controlled trial (RCT), (c) the article was written in English. The quality of evidence was evaluated according to the Grading of Recommendations, Assessment, Development and Evaluation (GRADE) approach. A meta-analysis was performed to determine effect sizes. Five studies were selected in the systematic review (162 participants) and four were included in the meta-analysis (142 participants). Results showed a significant enhancement in VO_2max_ in healthy adults and athletes (*p* = 0.04). The mean difference was 3.00 (95% CI from 0.18 to 5.82) with high heterogeneity. In conclusion, Ashwagandha supplementation might improve the VO_2max_ in athlete and non-athlete people. However, further research is need to confirm this hypothesis since the number of studies is limited and the heterogeneity was high.

## 1. Introduction

Maximum oxygen consumption (VO_2max_) is a physiological parameter that defines the aerobic capacity of a person. It is an indicator of the cardiorespiratory fitness that describes health status [1] and sport performance [2]. Focusing on competitive sports, the VO_2max_, together with running economy and the anaerobic threshold, is one of the main factors that determine success in endurance activities [3], and also contributes to increase the team sports performance by increasing work intensity, distance covered, and number of sprints completed [4]. However, from the point of view of the physical training, there are still controversies about the best training intensity to enhance the VO_2max_ [5,6]. 

Apart from sport performance, VO_2max_ has special interest in the field of health. Low values of VO_2max_ (<17.5 mL·min^-1^·kg^-1^) are associated with an increased risk of mortality and loss of independent lifestyle in adults and elderly [7], while high values of cardiorespiratory fitness have been associated with a reduced risk of cardiovascular diseases [8,9]. The VO_2max_ level is also important in children, where a higher aerobic capacity is related to better quality of life [10].

Ashwagandha (*Withania somnifera)* is a plant in the Solanaceae family. The extract of the Ashwagandha root has many biological implications due to its diverse phytochemicals [11], so it has been used, singly or in combination with other natural plants, in many research studies for its properties: anti-diabetic [12], anti-inflammatory [13], anti-microbial [14], anti-tumor [15], anti-stress [16], cardioprotective [17], or neuroprotective [18]. It also displays enhanced endothelial function [11], reduces reactive oxygen species [13], regulates apoptosis [19], and modulates mitochondrial function [11], showing to be effective to treat aging effects [20], anxiety and stress [21], arthritis [22], cognitive functions and memory [23], diabetes [12], epilepsy [24], fatigue [25], neurodegenerative diseases [26], pain [27], thyroid function [28], and skin diseases [29].

In spite of the relevant benefits of supplementation with Ashwagandha, only four meta-analyses have been carried out evaluating its efficacy on anti-inflammatory effects [30], on impotence and infertility treatment [31], on neurobehavioral disorders [32] and anxiety [33]. However, there are no meta-analyses that analyze the effect of Ashwagandha on physical performance. Therefore, the purpose of this study was to systematically review the scientific literature about the effects of supplementation with Ashwagandha on VO_2max_ and to provide practical recommendations. Besides, a meta-analysis was carried out to determine the effect sizes of Ashwagandha on VO_2max_.

## 2. Methods

The review was conducted following the statements of the Preferred Reporting Items for Systematic Reviews and Meta-Analyses Guidelines (PRISMA).

### 2.1. Literature Search

To find the studies reported in the meta-analysis, several electronic databases were screened: PubMed (Medline), Web of Science (which includes other databases such as Current Contents Connect, Derwent Innovations Index, Korean Journal Database, Medline, Russian Science Citation Index, and Scielo Citation Index) and Google Scholar. The search was conducted in September 2019. The search terms were: (a) the type of treatment (Ashwagandha or “*withania somnifera*”) and (b) the outcome variable (“oxygen consumption” or “aerobic” or “VO_2_”). The search was conducted using the treatment and the outcome variables, separated by the Boolean operator “and”.

### 2.2. Study Selection

The inclusion criteria were: (a) VO_2max_ data, with means ± standard deviation (SD) before and after the supplement intervention; (b) the study was a randomized controlled trial (RCT); (c) the article was written in English. Two independent authors selected the potentially eligible articles from the databases. There were no disagreements.

### 2.3. Quality of the Evidence and Risk of Bias

The quality of the evidence was categorized using the Grading of Recommendations, Assessment, Development and Evaluation (GRADE) approach. The risk of bias was assessed by the Cochrane Collaboration’s tool for assessing risk of bias. This tool classified the selection, performance, detection, attrition, and reporting bias into low, high, or unclear risk of bias.

### 2.4. Data Collection

Two authors independently extracted data from the studies. The information included: participants, interventions, comparisons, outcomes, and study design (PICOS), following the recommendations from the PRISMA statement. Table 1 shows age, sex, sample size, and condition of the participants. Table 2 presents intervention and the comparison groups, including type of supplementation with the doses, duration of the study, and the daily frequency of the supplementation. Figure 3 displays results for the different outcomes. Study design was not included in any table because all studies were RCT.

### 2.5. Statistical Analysis

The main outcome of this meta-analysis was VO_2max_. The meta-analysis was conducted using the Revision Manager (RevMan) software (version 5.3) obtained from Cochrane Collaboration web. Post-intervention mean and SD were extracted and used for meta-analyses. All articles reported VO_2_ max as mL/kg/min. Mean difference was calculated using a random model. The heterogeneity between the studies was calculated using Tau^2^, I^2^, and Chi^2^ tests. Although there is no consensus about the definition of “mild”, “moderate”, or “severe” heterogeneity, Higgins and Thompson [34] suggested that values for I^2^ higher than 56% would mean large heterogeneity while values lower than 31% would be related to low heterogeneity.

## 3. Results

### 3.1. Study Selection

**Figure 1:**
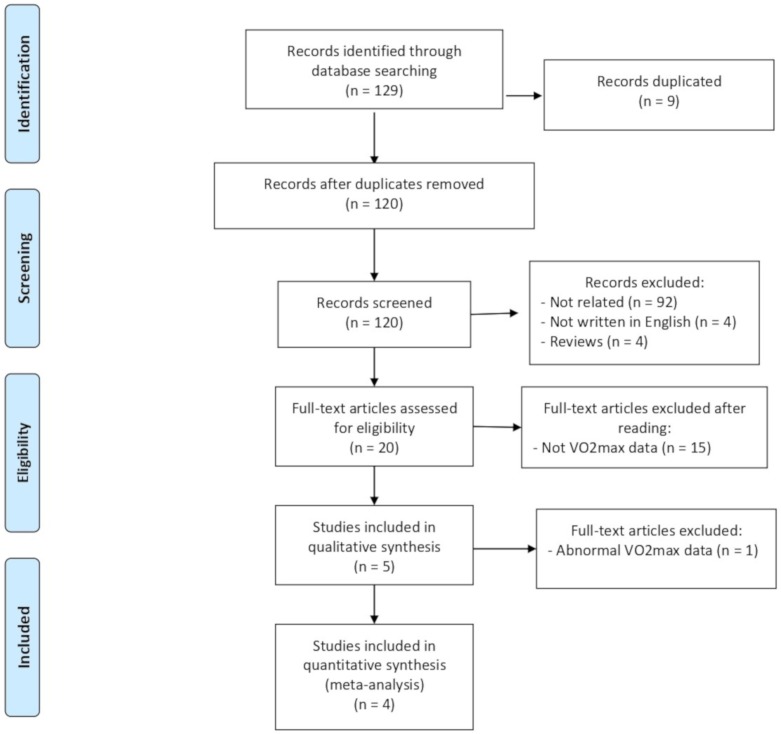
Flow chart delineating the complete systematic review process.

The PRISMA flow diagram is showed in Figure 1. A total of 129 records were identified, 9 of which were removed because they were duplicated. Of the remaining 120 articles, 92 were excluded because they were not related with the topic, 4 studies were not written in English, and 4 were reviews. After reading the remaining 20 articles, another 15 studies did not meet the inclusion criteria and were excluded. Therefore, 5 studies were included in the systematic review. However, the article by Sandhu et al. [35] was excluded from meta-analysis due to the odd results. In this regard, they evaluated healthy young males and females aged between 18 and 25 with body mass index between 18 and 25. Their mean peak VO_2max_ was lower than 14mL/kg/min, which is so much lower than expected for healthy young people and less than half the mean of the rest of the included studies (46.18 mL/kg/min). We tried to contact with the authors in order to obtain a reason for that, but at the time this article was considered for publication, we did not receive a response. Considering that in the article authors did not explain an incremental test to obtain the VO_2max_, we believe that they measured the gas exchange at rest, reporting the oxygen consumption (VO_2_). Therefore, this article was included in systematic review but not in the meta-analysis.

### 3.2. Quality of Evidence and Risk of Bias

**Figure 2:**
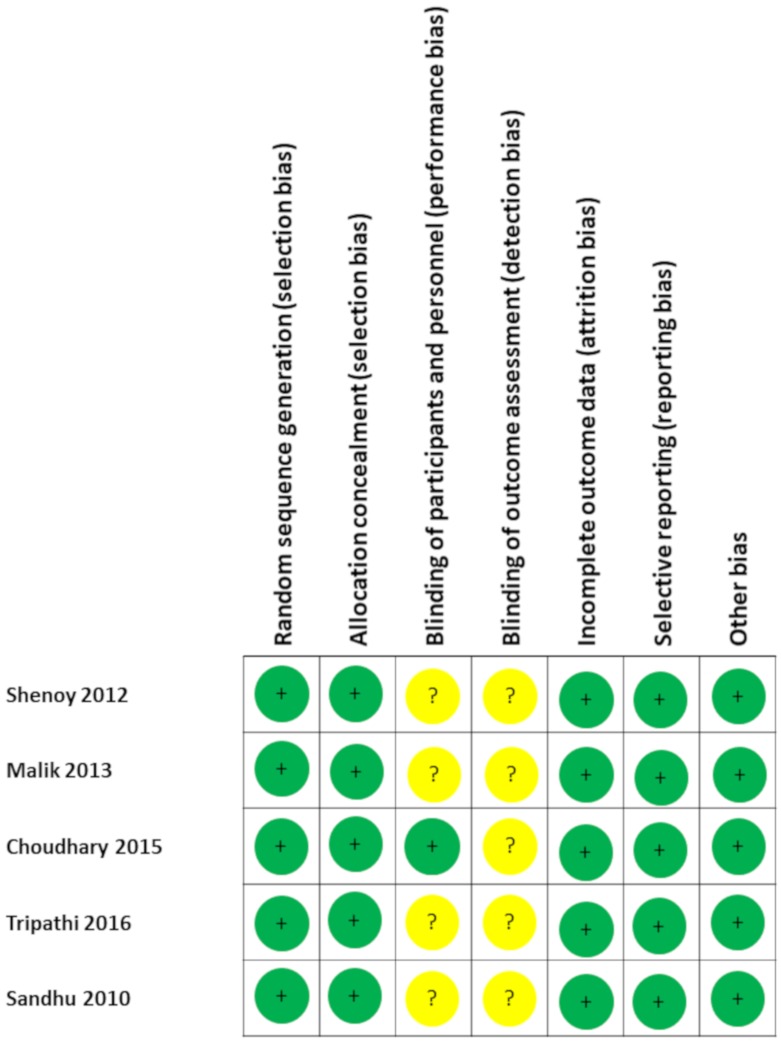
The Cochrane Collaboration’s tool for assessing risk of bias.

The evidence of the effects on VO_2max_ was initially classified as “high quality” due to all the selected articles were RCT, but the evidence dropped twice because of the small sample size and due to the high degree of heterogeneity. Therefore, the final quality of the evidence was low. The Cochrane Collaboration’s tool for assessing risk of bias (Figure 2) showed that the poorer scores were obtained in the performance and detection bias due to unclear reporting.

### 3.3. Study Characteristics

Study characteristics are summarized in Table 1. The total number of participants included in this systematic review were 162. Of these, 81 belonged to the Ashwagandha group and 81 were the placebo (control) group. The age ranged from 16 to 45 years old. The sample was comprised exclusively of healthy adults and athletes. 

### 3.4. Interventions

The characteristics of the Ashwagandha supplementation and placebo group are displayed in Table 2. The doses varied from 300 to 500 mg and the daily frequency intake was once or twice a day. The total duration of the intervention varied from 2 to 12 weeks.

### 3.5. Outcome Measures

The study of Choudhary et al. [36] found a significant group*treatment interaction in the VO_2max_. The remaining four articles only found within-group improvement in VO_2max_ after the supplement intervention [35,37,38,39].

**Figure 3:**
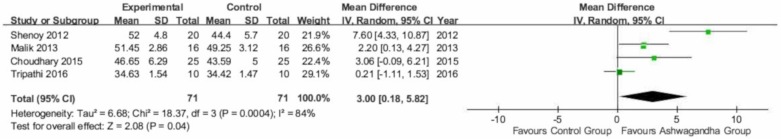
Meta-analysis results of the effects of Ashwagandha supplementation on VO_2max_.

Regarding meta-analysis results, a significant (*p* = 0.04) mean difference was observed. Figure 3 showed a mean difference of 3.00 (95% CI from 0.18 to 5.82). The heterogeneity level was large according to the I^2^ = 84%. The quality of the evidence was low according to the GRADE classification.

## 4. Discussion

The purpose of this study was to systematically review the scientific literature about the effects of supplementation with Ashwagandha on VO_2max_ and to carry out a meta-analysis to determine the overall effect. After 20 articles were assessed for eligibility, 15 articles were excluded since they did not report VO_2 max_. A total of 5 articles were included in the systematic review [35,36,37,38,39]. However, one article was excluded from the meta-analysis [35] since the reported mean VO_2max_ was abnormally low for healthy young people and less than half the mean of the rest of the included studies (46.18 mL/kg/min), which may indicate that they were not actually reporting VO_2max_ but VO_2_ at rest. The results of this meta-analysis showed that supplementation with Ashwagandha may be useful to improve VO_2max_ in athletes [36,38,39] and healthy adults [37]. Table 2 displayed the amount of Ashwagandha used in each study, which varied from 330 up to 1000 mg/day, which is inside the limits, 750 to 1250 mg/day, found to be well tolerated and safe [40]. In this regard, none of the five articles reported any relevant side effect as a consequence of the treatment, achieving a high compliance with the treatment and very low number of dropouts.

The two studies that achieved the highest treatment effect and effect size [36,39] were those with the highest Ashwagandha intake (>50 g in the whole program). Therefore, it seems like the higher the dose, the higher the improvement in VO_2_. However, the study by Tripathi, Shrivastava, Ahmad Mir, Kumar, Govil, Vahedi, and Bisen [14] did not observe any significant difference between the effects of a 330 mg intake and the effects of a 500 mg intake after 2 weeks. Therefore, further studies comparing the effect of different doses, as well as studies with longer duration are needed.

In general terms, the overall effects were better in those studies with a sample comprised of athletes [36,38,39] compared with the studies with healthy adults [14,39]. This is interesting since, as expected, baseline levels were higher in athletes and, consequently, larger improvements were expected in non-athlete healthy adults. It could be that the effects of supplementation with Ashwagandha might be linked to the physical activity levels of the participants, promoting and increasing the physiological adaptations to physical exercise. However, this hypothesis should be explored in future studies. The VO_2max_ defines the body’s ability to transport and utilize oxygen, so this physiological parameter is associated with endurance performance. Many factors contribute to the VO_2max_ values, including genetic predisposition [41], enzymes [42], muscle fiber type [43], or training [44]. It is also known that nutritional supplementation can improve the effects of training and reach higher performance [45]. Previous studies with Ashwagandha administration observed improvement in working capacity test in rats by increasing the swimming endurance test [46]. As endurance performance is determined by mitochondrial function, some reasons for the Ashwagandha to improve cardiorespiratory fitness can be the significant effects observed on mitochondrial and energy levels, by reducing the succinate dehydrogenase enzyme activity in the mitochondria and benefiting Mg-ATPase activity [47]. Previous studies showed that Ashwagandha significantly enhanced the hemoglobin concentration and red blood cells in animals [48] and also in humans [38], with the subsequent increase in the capacity to transport oxygen to the muscles. Moreover, it should be considered that Ashwagandha has shown to have anti-fatigue [49,50] and anti-stress [51] actions. This could be connected to the significant improvement in the time to exhaustion of the experimental group that could be observed in the study of Shenoy, Chaskar, Sandhu, and Paadhi [39]. Some of the chemical constituents of *Whitania somnifera* [52] such as flavonoids, alkaloids, and steroidal lactones (withanolides) or the antioxidants (superoxide dismutase, catalase, and glutathione peroxidase) could be behind the improvements of VO_2max_. Therefore, further studies are needed to explore which are the chemical constituents and mechanism that may explain the potential improvement in the VO_2max_.

Although all mechanisms by which Ashwagandha can improve the VO_2max_ have not been described yet and future studies are needed to elucidate that improvement, it is known that Ashwagandha exhibits little or no associated toxicity [53], so it seems that this Ayurvedic herb “Ashwagandha” (*Withania somnifera*) can be safely used for improving cardiovascular fitness in healthy adults and also in athletes, offering an additional alternative as a nutritional supplement to enhance VO_2max._

Some limitations in the present meta-analysis can be mentioned. The first one is related to the search strategy, only articles published in English were included and a few databases were used. Another limitation can be the large heterogeneity in the included articles. Different doses, levels of physical activity, or the inclusion of both women and men in the protocols make it very difficult to achieve a high level of evidence. In addition, the systematic review and meta-analysis was not prospectively registered in any public database. Furthermore, in order to have a better understanding of long-term ergogenic benefit and potential side effects from Ashwagandha root extract, longer duration studies are needed.

## 5. Conclusions

Ashwagandha supplementation might improve the VO_2max_ in athlete and non-athlete people. The analyzed studies used oral administration of Ashwagandha which varied between 2 and 12 weeks with intakes between 300 to 1000 mg/day. Due to the limited number of studies included in this systematic review and meta-analysis, further research is needed to confirm the effects and the recommended dose.

## Figures and Tables

**Table 1:** Characteristics of the sample.

RCT	Weeks	Groups, Sample Size and Sex	Age (Years)	Country	Population
Shenoy 2012	8	AS: 20 (M and F) CG: 20 (M and F)	18–27	India	Elite cyclists
Malik 2013	8	AS: 16 (M) CG: 16 (M)	16–19	India	Hockey players
Choudhary 2015	12	AS: 25 (M and F) CG: 25 (M and F)	20–45	India	Athletes
Tripathi 2016	2	AS: 10 (M) CG: 10 (M)	18–45	India	Healthy adults
Sandhu 2010	8	AS: 10 (M and F) CG: 10 (M and F)	18–25	India	Healthy adults

RCT: randomized controlled trial; AS: Ashwagandha group; M: males; F: females; CG: control group.

**Table 2:** Characteristics of the interventions.

RCT	Ashwagandha Group	Control Group	Dose (mg)	Duration of the Study	Daily Frequency	Total Dose (g)
	Type of Supplementation	Type of Supplementation				
Shenoy 2012	Ashwagandha in gelatin capsules	Capsules containing starch powder	500	8 weeks	twice	56
Malik 2013	Roots of WS	Sugar power was filled in gelatin capsules	500	8 weeks	once	28
Choudhary 2015	One capsule of KSM-66 Ashwagandha	Identical capsules containing sucrose	300	12 weeks	twice	50.4
Tripathi 2016	WS aqueous extract in the capsule form	Maize starch capsule	330	2 weeks	once	4.62
Sandhu 2010	WS filled in gelatin capsules	Capsules filled with flour	500	8 weeks	once	28

RCT: randomized controlled trial; KSM-66: commercial name of an Ashwagandha extract; WS: Withania Somnifera. Total dose was calculated as: total dose (g) = (dose (mg) × daily frequency × study duration (days))/1000.
